# The Treatment Status of Patients in NSCLC With RET Fusion Under the Prelude of Selective RET-TKI Application in China: A Multicenter Retrospective Research

**DOI:** 10.3389/fonc.2022.864367

**Published:** 2022-05-24

**Authors:** Yan Meng, Yilin Yang, Yujia Fang, Xinqing Lin, Xiaohong Xie, Haiyi Deng, Jianhui Wu, Maolin Zhou, Ni Sun, Zhanhong Xie, Ming Liu, Ming Ouyang, Yinyin Qin, Chunxia Su, Chengzhi Zhou

**Affiliations:** ^1^ State Key Laboratory of Respiratory Disease, National Clinical Research Centre for Respiratory Disease, Guangzhou Institute of Respiratory Health, First Affiliated Hospital, Guangzhou Medical University, Guangzhou, China; ^2^ Ward 2, Department of Oncology, Hainan Cancer Hospital, Haikou, China; ^3^ Department of Medical Oncology, Shanghai Pulmonary Hospital & Thoracic Cancer Institute, School of Medicine, Tongji University, Shanghai, China

**Keywords:** RET, NSCLC, tyrosine kinase inhibitors, survival, risk factor

## Abstract

**Background:**

Rearranged during transfection (RET) fusion is a kind of uncommon mutation (about 1%) in non-small cell lung cancer (NSCLC). Although selective tyrosine kinase inhibitors (TKI) (selpercatinib and pralsetinib) have been available, there are no real-world data about the difference in the efficacy between RET-TKI and other regimens in China.

**Methods:**

We conducted a multicenter retrospective analysis of 49 patients with RET-fusion-positive NSCLC. The characteristics and the clinical outcomes with RET-TKI, multi-kinase inhibitor (MKI), systematic chemotherapy, and immune-checkpoint inhibitor (ICI)-based regimens were evaluated.

**Results:**

Of the 92 treatments in patients included, RET-TKI was administered 24 times (26.1%), systematic chemotherapy was 35 times (38.0%), ICI-based regimens was 26 times (28.3%), and MKI was 7 times (7.6%). RET-TKI had a higher objective response rate than the chemotherapy and ICI-based regimens (63.6% vs. 14.3% vs. 21.0%, p < 0.001). The median progress-free survival (mPFS) of RET-TKI, chemotherapy, immunotherapy, and MKI was 16.9 (95% CI: 1.8–32.0) months, 11.9 (95% CI: 7.7–16.1) months, 6.7 (95% CI: 2.9–10.5) months, and 2.8 (95% CI: 1.1–4.4) months, respectively. The mPFS of RET-TKI was longer than MKI and immunotherapy (p < 0.001), while without difference with chemotherapy (p = 0.096). Moreover, chemotherapy had longer mPFS than MKI (p < 0.001). In subgroup analysis, patients with brain metastases in RET-TKI treatment had worse mPFS than the one of patients without brain metastases (6.1 (95% CI: 0.0–13.9) months and 8.5 (95% CI: 6.3–10.6) months, p = 0.012). For patients having chemotherapy with or without angiogenesis inhibitors, the mPFS was 12.0 (95% CI: 11.05–13.02) months and 9.1 (95% CI: 8.31–9.89) months (p = 0.468). In the group of ICI-based regimens, the expression level of PD-L1 did not affect the mPFS of ICI [PD-L1 (+) vs. PD-L1 (–): 4.7 (95% CI: 1.8–9.0) months vs. 7.6 (95% CI: 1.1–14.0) months, p = 0.910]. For overall patients, ECOG PS score, therapy lines, and therapeutic regimens were the independent factors affecting the prognosis.

**Conclusions:**

In RET-fusion-positive NSCLC, RET-TKI is the best choice for a better response rate and PFS. In addition, chemotherapy which may bring a good PFS, is still a good choice for this group of patients.

## Introduction

Rearranged during transfection (RET) is a kind of transmembrane receptor tyrosine kinase, which plays an important role in the early development of kidneys and the enteric nervous system. With proto-oncogene properties, RET associates with cell proliferation, growth, differentiation, and survival through activation of downstream signaling pathways such as RAS/MAPK, PI3K/AKT, and JAK/STAT (1, 2). RET aberration mainly has two forms of mutation, namely, point mutations and fusions. The former is more related to the occurrence and development of medullary thyroid cancer, while the latter is more related to papillary thyroid cancer and non-small cell lung cancer (NSCLC) (3).

First found in 2012, RET fusion is one of the rare gene mutations in NSCLC (about 1%–2%) (4, 5). Although the incidence is low, basing on the huge base of the NSCLC population, it is worth to study the characteristics, prognosis, and the treatments, which could bring better efficacy of this group of patients. Over the past decade, the treatment of RET-fusion-positive NSCLC patients has evolved from chemotherapy alone to multi-kinase inhibitor (MKI) to selective RET (tyrosine kinase inhibitors) (RET-TKI) nowadays. In particular, due to the excellent efficacy results of RET-TKIs, they were quickly approved for indications in just 2 years and became the first-line treatment recommendation for patients with RET-fusion-positive NSCLC in the National Comprehensive Cancer Network guidelines. Chinese Society of Clinical Oncology guidelines also list selpercatinib as a level III recommendation for RET-fusion-positive patients no matter in any treatment line; pralsetinib as a level II recommendation for subsequent-line treatment.

Although the efficacy of the regimens except RET-TKIs is limited, they are still a reasonable choice for NSCLC patients with RET fusion, especially under the prelude of the RET-TKI application, meaning that issues such as not only price but also accessibility may turn many patients away (6). According to previous data, other treatments such as chemotherapy can also bring efficacy and can still be the treatment options for this population. However, there is lack of study to directly compare the efficacy of different regimens in the real world.

In order to bring more data support for the better choice of regimens for NSCLC patients with RET fusion, we study the efficacy of RET-TKI in the real world and explore the difference between other treatment options and RET-TKI, including MKI, chemotherapy, and immune-checkpoint inhibitor (ICI)-based regimens.

## Materials and Methods

### Study Design and Patients

We conducted a retrospective study of all patients with RET+ NSCLC in three centers (center 1: Hainan Cancer Hospital, center 2: the First Affiliated Hospital of Guangzhou Medical University & State Key Laboratory of Respiratory Disease, center 3: Shanghai Pulmonary Hospital & Thoracic Cancer Institute) from January 2015 to December 2021. The selected patients must be NSCLC patients with RET rearrangement at the time of initial diagnosis and had a specific treatment history (including regimens, the time of start of use, the time of end of use, and the reason of discontinuation). The patients who acquired other treatable mutations such as EGFR mutation were excluded due to the concern of the potential prognostic impact of other TKI administration.

### Data Collection

The baseline information at the time of diagnosis of included patients was collected including age, sex, smoking history, history of lung disease, Eastern Cooperative Oncology Group Performance Status score (ECOG PS) score, histologic types, tumor node metastasis (TNM) stage, with or without brain metastases, RET fusion partner, and the expression of programmed cell death ligand 1 (PD-L1) of patients using ICIs. The histologic type was based on the fifth edition of the WHO classification of lung tumors. The TNM stage was classified according to the eighth edition of the TNM Classification for Lung Cancer (6). RET fusion was detected locally at each center and collected retrospectively. Detection methods include next-generation sequencing and reverse transcription-polymerase chain reaction. The expression of PD-L1 was assessed by immunohistochemistry using the 22C3 antibody. When the expression <1%, it was recorded as negative PD-L1 expression [PD-L1 (–)]; when the expression ≥1%, a positive PD-L1 expression [PD-L1 (+)] was recorded.

### Tumor Response Assessment

The information of treatment for each patient was recorded including treatment line, treatment regimen, efficacy, date of treatment beginning, progression or loss to follow-up or latest follow-up, and survival status. The specific treatment regimens were divided into four cohorts, namely, RET-TKI, MKI, chemotherapy, and ICI-based regimens, and their objective response rate (ORR) and progression-free survival (PFS) were set as the main outcomes. Since median overall survival time has not been reached, it was not included as one of the outcomes of this study. We performed further subgroup analyses of the efficacy of different treatments in first-line or subsequent-line treatments. Besides, in order to figure out the influence of brain metastasis on the efficacy of RET-TKI, a subgroup analysis of RET-TKI in patients with or without brain metastasis was performed. For the reason that the addition of angiogenesis inhibitors may affect the efficacy, we performed subgroup analyses of the efficacy of chemotherapy with or without angiogenesis inhibitors. For ICI-based regimens, whether the expression of PD-L1 would affect the efficacy was analyzed.

The efficacy assessment was based on the Response Evaluation Criteria in Solid Tumors (RECIST version 1.1), and the tumor response included complete response (CR), partial response (PR), stable disease (SD), and progressive disease (PD) (7). ORR was defined as the proportion of patients with tumor response in CR or PR to the total population. PFS was defined as the time from the beginning of treatment to disease progression or death.

### Statistical Analysis

Continuous variables are described by median (minimum to maximum), and categorical variables were described by frequencies (percentages). Differences in ORR between groups were achieved by the chi-square test, and the Z test was used for pairwise comparisons. The median PFS and its 95% confidence interval (CI) were obtained through the Kaplan–Meier method, and the log-rank test and Breslow test were used to compare survival curves.

Age, sex, smoking history, ECOG PS score, brain metastasis, RET fusion partner, treatment regimens, and treatment line were included in the risk factor analysis of PFS. We used the Cox regression model to do the univariate survival analysis and multivariable survival analysis. If the p-value is less than 0.1 in the univariate survival analysis, the factors would be included in the multivariable survival analysis. A hazard ratio (HR) with 95% CI and its p-value was used to describe the results. Except for special instruction, a two-sided p value of less than 0.05 (p < 0.05) was considered statistically significant. Bonferroni correction was used if there were more than two groups needed to be compared. Statistical analyses were conducted by SPSS version 25.0 (IBM Corporation, Armonk, NY, USA), while data were visualized with GraphPad Prism version 8.0.0 for Windows (GraphPad Software, San Diego, CA, USA).

## Results

### Patient Characteristics

A total of 49 patients with RET-rearranged NSCLC were included in this study (15 in center 1, 10 in center 2, 24 in center 3). The median age of the included patients was 56 (range: 26–77). The distribution of men (26, 53.1%) and women (23, 46.9%) was almost equal. A percentage of 69.4% of patients never smoked. Except one who had sarcomatoid carcinoma, all patients were diagnosed with adenocarcinoma. A percentage of 20.8% of patients had brain metastases at initial diagnosis. As for the gene-fusion spectrum, KIF5B was the most common RET fusion partner, while others included CCDC6, NCOA4, and TXNDC11. The specific information of patient characteristics is shown in **Table 1**.

**Table 1:** The baseline information of patients at the time of diagnosis.

Characteristics	Patients (n = 49)
Age—y Median Range	56 26–77
Male/female—no. (%)	26 (53.1%)/23 (46.9%)
Smoking status—no. (%) Former/current Never	15 (30.6%) 34 (69.4%)
Histologic types—no. (%) Adenocarcinoma Sarcomatoid carcinoma	48 (98.0%) 1 (2.0%)
TNM stages—no. (%) IIIA IIIB IIIC IVA IVB	1 (2.0%) 1 (2.0%) 2 (4.2%) 23 (46.9%) 22 (44.9%)
ECOG PS 0 1 2	1 (2.0%) 40 (81.6%) 8 (16.3%)
Brain metastasis Yes No Unknown	11 (22.4%) 37 (75.5%) 1 (2.0%)
RET fusion KIF5B CCDC6 NCOA4 TXNDC11 Unknown	13 (26.5%) 6 (12.2%) 1 (2.1%) 1 (2.1%) 28 (57.1%)

y, years old; TNM, tumor node metastasis; ECOG PS, Eastern Cooperative Oncology Group Performance Status; RET, rearranged during transfection.

### Efficacy of Overall Patients

**Figure 1:**
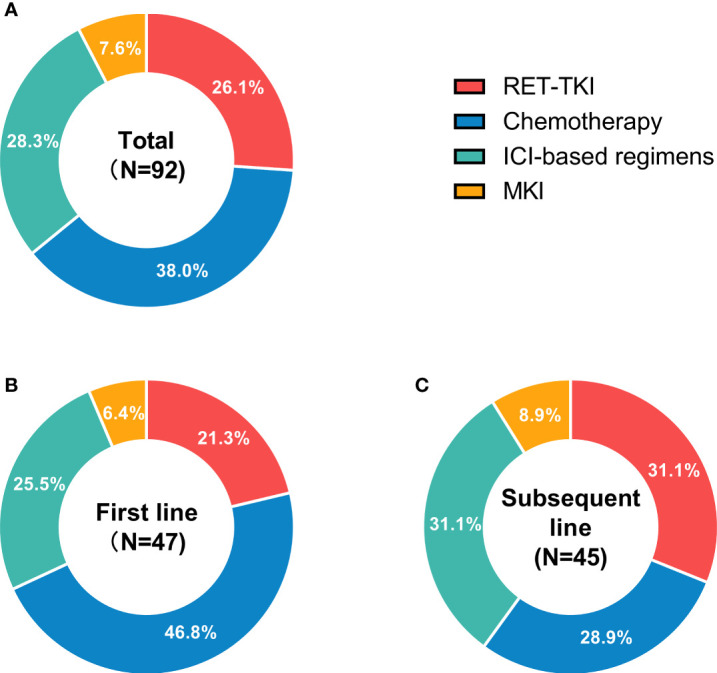
Status of different treatments for RET-fusion non-small cell lung cancer patients in different treatment-lines. **(A)** The usage status of different treatments in any treatment line. **(B)** The usage status of different treatments in first-line treatments. **(C)** The usage status of different treatments in subsequent-line treatments. N, usage count. RET-TKI, rearranged during transfection-tyrosine kinase inhibitors. ICI, immune-checkpoint inhibitor; MKI, multi-kinase inhibitor.

Of the 92 treatments in 49 patients, 24 had received RET-TKI, 7 for MKI, 35 for chemotherapy, and 26 for ICI-based regimens (**Figure 1A**). The specific regimens are shown in **Table 2**. The median follow-up time was 9.4 (95% CI: 6.8–12.0) months.

**Table 2:** Specific treatment regimens of included patients.

Treatment	Patients n (%)
RET-TKI Selpercatinib Pralsetinib	24 9 (37.5%) 15 (62.5%)
Chemotherapy Pemetrexed-based regimens Paclitaxel-based regimens Docetaxel Platinum	35 25 (71.4%) 7 (20.0%) 2 (5.7%) 1 (2.9%)
ICI-based regimens Anti-PD-1 Anti-PD-L1 Unknown	26 20 (76.9%) 2 (7.7%) 4 (15.4%)
MKI Cabozantinib Alectinib Lenvatinib	7 4 (57.1%) 2 (28.6%) 1 (14.3%)

RET-TKI, rearranged during transfection-tyrosine kinase inhibitors; ICI, immune-checkpoint inhibitor; PD-1, programmed cell death protein 1; PD-L1, programmed cell death ligand 1; MKI, multi-kinase inhibitor.

**Figure 2:**
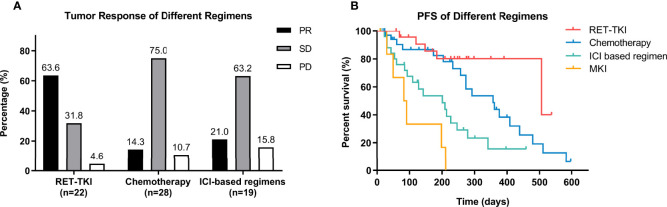
Efficacy analysis of different regimens in overall patients. **(A)** Tumor response of different regimens including RET-tyrosine kinase inhibitors (RET-TKI) or chemotherapy or immune-checkpoint inhibitor (ICI)-based regimens or multi-kinase inhibitor (MKI). **(B)** Progression-free survival (PFS) of patients treated with RET-TKI or chemotherapy or ICI-based regimens or MKI. PR, partial response; SD, stable disease; PD, progressive disease.

A total of 22 patients with RET-TKI, 1 with MKI, 28 with chemotherapy, and 19 with ICI-based regimens had information to assess the best tumor response. The ORRs of RET-TKI, chemotherapy, and immunotherapy-based regimens were 63.6% (95% CI: 41.8–85.5), 14.3% (95% CI: 0.5–28.1), and 21.0% (95% CI: 0.9–41.2), respectively (p < 0.001) (**Figure 2A**). As for pairwise comparison, the ORR of RET-TKI was better than that of chemotherapy or ICI-based regimens (p < 0.05), but there was no statistically significant difference between chemotherapy and ICI-based regimens.

All patients in any treatment regimen participated in the PFS analysis. RET-TKI had the longest median PFS (mPFS) (16.9 [95% CI: 1.8–32.0) months]. This was followed by chemotherapy [11.9 (7.7–16.1) months], ICI-based therapy [6.7 (95% CI: 2.9–10.5) months], and MKI [2.8 (95% CI: 1.1–4.4) months]. No matter in the log-rank test or Breslow test, the difference in mPFS of RET-TKI compared with ICI-based regimens or MKI and the difference between mPFS of chemotherapy and the one of MKI were statistically significant. In the comparison of chemotherapy and ICI-based regimens, the statistical difference existed only in the Breslow test, suggesting that there was a significant difference at the beginning, but the difference is no longer significant as time when on. The difference in median PFS between RET-TKI and chemotherapy was not statistically significant (**Figure 2B** and **Table S1**).

### Efficacy in Different Treatment Lines

Different regimens were used at different frequencies in each treatment line. In the first line, there were more patients used chemotherapy (46.8%), while in subsequent-line treatments, ICI-based regimens and RET-TKI were the main choices (**Figures 1B, C**).

**Figure 3:**
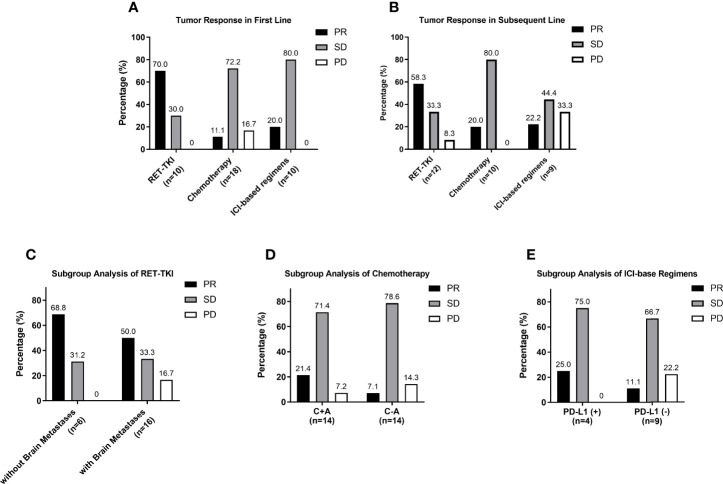
Subgroup analysis of tumor response. **(A)** Analysis of tumor response of different regimens in first-line treatment. **(B)** Analysis of tumor response of different regimens in subsequent-line treatment. **(C)** Analysis of tumor response in group of patients treated with RET-TKI with brain metastases or without. **(D)** Analysis of tumor response in group of patients treated with chemotherapy with or without angiogenesis inhibitor. **(E)** Analysis of tumor response in group of patients treated with ICI-based regimens with negative PD-L1 or positive PD-L1. PR, partial respons;. SD, stable disease; PD, progressive disease.

For the reason that the number of patients using MKI in each subgroup was too small be analyzed, the subgroup analysis did not include it. The ORR of RET-TKI, chemotherapy, and ICI-based regimens had a significant difference in first-line treatments (70% (95% CI: 34.8–93.3) vs. 11.1% (95% CI: 1.4–34.7) vs. 20.0% (95% CI: 2.5–55.6), p = 0.005) (**Figure 3A**). In pairwise comparisons, the difference only existed between RET-TKI and chemotherapy. Although a numerical difference is shown among different regimens in subsequent-line treatments [58.3% (95% CI: 27.7–84.8) for RET-TKI, 20.0% (95% CI: 2.5–55.6) for chemotherapy, 22.2% (95% CI: 2.8–60.0) for ICI-based regimens] (**Figure 3B**), there was no significant difference for these subgroup analyses (p = 0.146).

**Figure 4:**
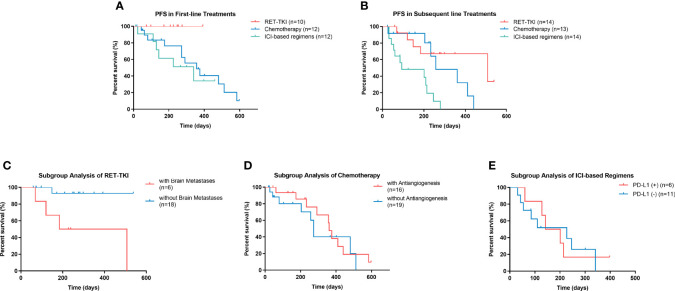
Progression-free survival (PFS) of patients with rearranged during transfection rearrangements (RET) in different conditions. **(A)** PFS of patients with RET-TKI, chemotherapy, or ICI-based regimens in first-line treatments. **(B)** PFS of patients RET-TKI, chemotherapy, or ICI-based regimens in subsequent-line treatments. **(C)** PFS of patients treated with RET-TKI with or without brain metastases. **(D)** PFS of patients treated with chemotherapy with or without angiogenesis inhibitors. **(E)** PFS of patients treated with ICI-based regimens with negative PD-L1 expression or positive one. PFS, progression-free survival, RET-TKI, rearranged during transfection-tyrosine kinase inhibitors. ICI, immune-checkpoint inhibitor. MKI, multi-kinase inhibitor; PD-L1, programmed death-ligand 1.

The mPFS of RET-TKI in first-line treatments had not been achieved, and its median follow-up time was 7.6 (95% CI: 5.6–9.6) months. For chemotherapy and ICI-based regimens, the median PFS in the first-line treatment was 11.9 (95% CI: 7.2–16.6) months and 11.4 (95% CI: 3.1–19.7) months, respectively. However, there was no significant difference among these three regimens (p = 0.527) (**Figure 4A**; **Table S2**). In subsequent-line treatments, RET-TKI [16.9 (95% CI: 7.9–32.4) months] and chemotherapy [8.6 (95% CI: 3.6–13.6) months] had longer mPFS than ICI-based regimens [3.0 (95% CI: 0.0–9.0) months] (p = 0.001 and p = 0.004) (**Figure 4B**; **Table S2**).

### Subgroup Analysis for Different Regimens

In a subgroup analysis for the RET-TKI group, six patients had brain metastases. The ORR and intracranial ORR were 50.0% (95% CI: 11.8%–88.2%) and 33.3% (95% CI: 4.3%–77.7%). However, there was no statistical difference in ORR with or without brain metastases (50.0% vs. 68.8%, p = 0.624) (**Figure 3C**). For chemotherapy, 16 patients used it with angiogenesis inhibitors (C+A) and 19 patients without (C-A). Although the ORR of C+A was numerically higher [21.4% (95% CI: 4.7%–50.8%) vs. 7.1% (95% CI: 0.2%–33.9%)], the difference was not statistically significant (p = 0.596) (**Figure 3D**). In the group of ICI-based regimens, a total of 13 patients had evaluated the expression of PD-L1, among which the ORR of patients with a negative PD-L1 expression was 11.1% (95% CI: 0.3%–48.2%), and the ORR of patients with a positive PD-L1 expression was 25.0% (95% CI: 0.6%–80.6%) (p = 1.000) (**Figures 3E**).

The mPFS of patients with brain metastases in the RET-TKI group was 6.1 (95% CI: 0.0–13.9) months; the mPFS of patients without brain metastases was not reached [the median follow-up time was 8.5 (95% CI: 6.3–10.6) months] (p = 0.012) (**Figure 4C**). The mPFS was 12.0 (95% CI: 11.1–13.0) months for C+A and 9.1 (95% CI: 8.3–9.9) months for C-A, but the difference was not significant (p = 0.468) (**Figure 4D**). For ICI-based regimens, the expression of PD-L1 did not bring a statistically significant difference for mPFS [4.7 (95% CI: 1.8–9.0) months vs. 7.6 (95% CI: 1.1–14.0) months, p = 0.910] (**Figure 4** **E**).

### Risk Factor Analysis

**Figure 5:**
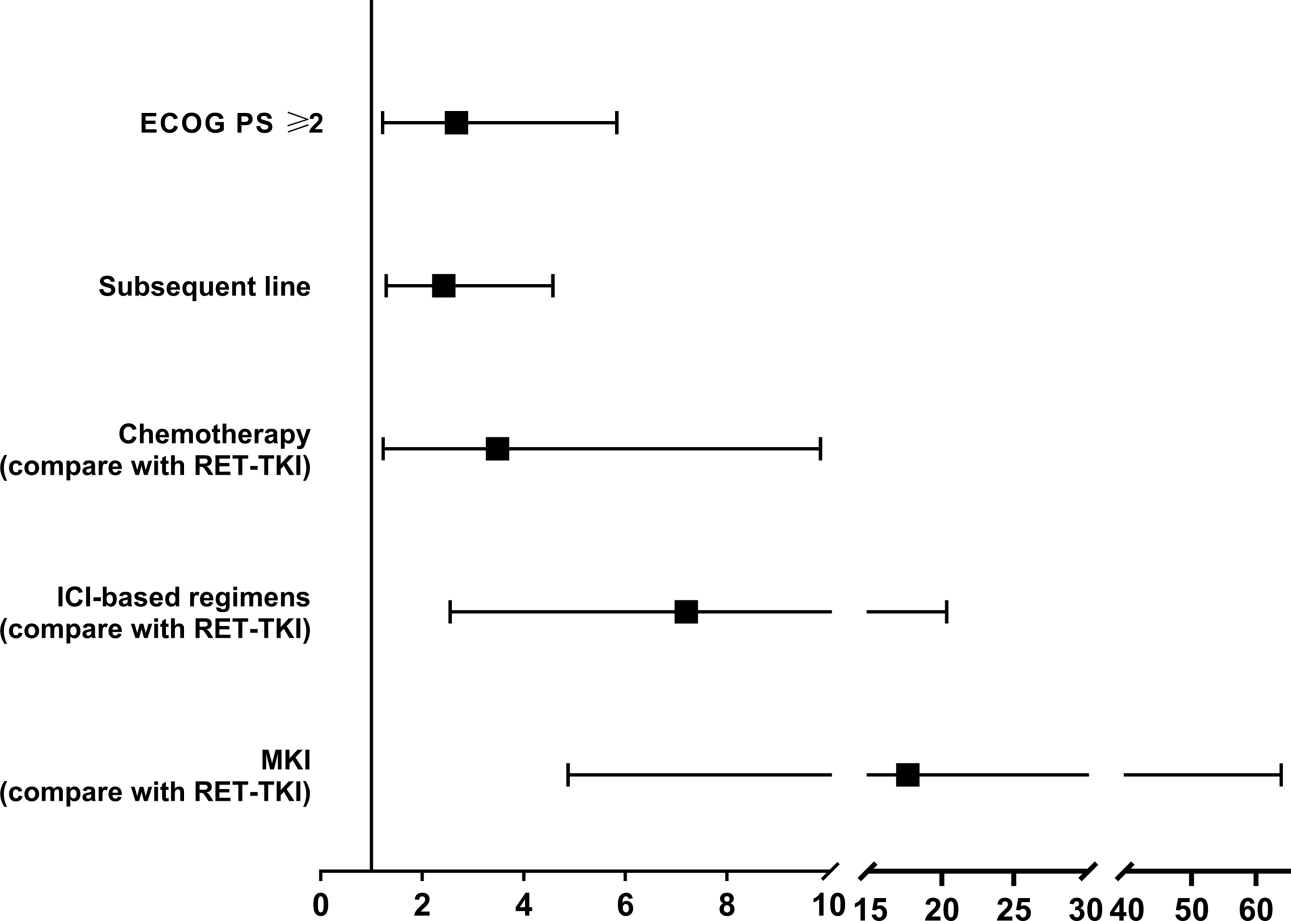
The forest plot of the risk factors associated with the efficacy of patients with RET-fusion positive non-small cell lung cancer. RET-TKI, rearranged during transfection-tyrosine kinase inhibitors. ICI, immune-checkpoint inhibitor; MKI, multi-kinase inhibitor; PD-L1, programmed death-ligand 1.

In the overall population, age<60, ECOG PS score ≥2, brain metastases, subsequent-line treatment, and chemotherapy, immunotherapy, and MKI compared with RET-TKI were associated with worse PFS in the univariate analysis. In the multivariate analysis, the p-value of ECOG PS score ≥2 [HR: 2.672 (95% CI: 1.224–5.834)], subsequent line treatment [HR: 2.42 (95% CI: 1.29–4.57)], and treatments other than RET-TKI (chemotherapy (HR: 3.48 (95% CI: 1.23–9.84), ICI-based regimens [HR: 7.20 (95% CI: 2.55–20.34), MKI (HR: 17.63 (95% CI: 4.87–63.87)] were less than 0.05, suggesting that the above factors were independent risk factors for poor PFS (**Figure 5**; **Table 3**).

**Table 3:** Univariate and multivariate Cox regression analyses for PFS in overall patients.

Factors	Univariate analysis		Multivariate analysis	
	HR (95% CI)	p-value	HR (95% CI)	p-value
Age (years) <60 ≥60	1 0.457 (0.228–0.916)	0.027		0.384
Sex Male Female	1 0.713 (0.392–1.297)	0.267		
Smoking history No Yes	1 0.708 (0.366–1.367)	0.304		
ECOG PS 0–1 2	1 3.543 (1.734–7.239)	0.001	1 2.672 (1.224–5.834)	0.014
Brain metastases No Yes	1 1.763 (0.977–3.181)	0.060		0.779
RET fusion partner KIF5B Others	1 1.097 (0.431-2.793)	0.846		
Treatment line First Subsequent	1 1.809 (1.009–3.244)	0.047	1 2.423 (1.286–4.567)	0.006
Treatment regimens RET-TKI Chemotherapy ICI-based regimens MKI	1 2.362 (0.872–6.393) 5.581 (2.050–15.196) 15.054 (4.401–51.495)	0.091 0.001 <0.001	1 3.478 (1.232–9.842) 7.198 (2.547–20.340) 17.628 (4.865–63.873)	0.019 <0.001 <0.001

HR, hazard ratio; ECOG PS, Eastern Cooperative Oncology Group Performance Status score; RET-TKI, rearranged during transfection-tyrosine kinase inhibitors; ICI, immune-checkpoint inhibitor; MKI, multi-kinase inhibitor.

## Discussion

In this multicenter retrospective research of RET-fusion-positive NSCLC patients, we described the clinical characteristics and compared the efficacy among the latest treatment regimens including RET-TKI, chemotherapy, ICI-based regimens, and MKI. The results support that RET-TKI is the first choice of NSCLC patients with RET fusion, while chemotherapy especially with angiogenesis inhibitors is still a good choice. Similar with the former studies, the efficacy brought by ICI and MKI was limited. In our knowledge, this is a comparative efficacy study to date that includes the latest and most comprehensive treatment options for patients with RET-fusion-positive NSCLC in the real world. Moreover, the findings from this study can give advices for the better clinical decision making.

TKI of other gene mutations such as EGFR and ALK has brought long survival to patients harboring corresponding mutations. However, patients with a RET-fusion mutation have not been able to obtain a good prognosis until the emergence of two RET-TKIs, selpercatinib and pralsetinib, in 2018 (8). The phase 1–2 clinical trial LIBRETTO-001 of selpercatinib showed that the objective response (OR) was 64% for patients previously receiving at least platinum-based chemotherapy and 85% for treatment-naïve patients, while the median PFS was 16.5 (95% CI: 13.7 to NE) months and not reached, retrospectively (9). For the Chinese population, a further phase 2 study LIBRETTO-321 was conducted by Lu et al. Similar to LIBRETTO-001, the ORR was 66.0% with 96.8% of responses ongoing at a median follow-up of 10.3 months, further demonstrating the stable efficacy of selpercatinib (10). Another phase 1–2 clinical trial ARROW of pralsetinib also achieved a good outcome, showing 61% OR for patients treated in subsequent lines and 70% for patients treated in the first line; the mPFS was 16.5 months for patients after treatment and 13.0 months for treatment-naïve patients (11). Based on far better efficacy and safety than other previous treatments, the FDA approved selpercatinib and pralsetinib for adult patients with metastatic RET fusion-positive NSCLC in 2020, while the National Medical Products Administration of China granted accelerated approval for pralsetinib in 2021. The overall results of our study also showed a good efficacy of RET-TKI with 63.6% ORR and a 16.9-month mPFS. The ORR of RET-TKI was 58% in subsequent-line treatments and 70% for treatment-naïve patients. The mPFS was 16.9 months for patients after treatment, and the one for patients in first-line treatments was not reached. These data are very close to the results of clinical trials. However, the difference between RET-TKI with chemotherapy or ICI-based regimens seems to just exist in subsequent-line treatments. As the follow-up time of patients with RET-TKI was not long enough, and the number of patients was limited, the results still need further follow-up data to certify.

For the reason that RET mutation is the risk factor for brain metastasis (12), and the incidence of brain metastasis during the lifetime of patients with RET fusion is nearly 50% (13), the intracranial response of RET-TKIs is one of the focuses. Both selpercatinib and pralsetinib showed good intracranial efficacy in clinical trials. In LIBRETTO-001, 22 patients with measurable intracranial disease at baseline achieved 82% ORR including 23% with CR. In overall patients, the median intracranial PFS was 13.7 months (14). In the ARROW study, four in eight patients with brain metastases at the time of diagnosis obtained OR, with two CRs (11). All these results show that both two RET-TKIs can cross the blood–brain barrier and bring good efficacy. Among 24 patients treated with RET-TKI in this study, 25% patients had brain metastases when diagnosed. According to the mPFS of subgroup analysis, brain metastases were an independent risk factor for a shorter time to RET-TKI benefit although the mPFS still had more than 6 months. Among the six patients, the intracranial ORR was 33.3% (all had CR), which seems lower than the results of clinical trials. This bias may be related to the heterogeneity caused by the small number of patients. At the same time, different RET-TKIs may also affect the results as the intracranial efficacy of selpercatinib seems to be better as seen in clinical trials. However, there will not be any head-to-head comparison, and a meta-analysis may be helpful to find out the detailed differences between the two RET-TKIs when clinical trial data gradually increase in the future. Besides, a real-world study with larger numbers of patients with brain metastases is needed to further evaluate the intracranial efficacy of RET-TKIs.

Before the development of RET-TKIs, chemotherapy was the recommendation of first-line therapy. A retrospective study from Drilon et al. showed that the mPFS of 18 RET-rearranged lung cancer patients was 19 months (15), proving that RET-rearranged patients could also benefit from pemetrexed-based systemic therapies. Besides, a study from China also proved that pemetrexed-based chemotherapy is better than other chemotherapy regimens (mPFS: 9.2 vs. 5.2 months) (16). In our study, the mPFS of chemotherapy is similar to the former studies. Although it was shorter than the one of RET-TKI numerically, the difference did not have a statistical significance. We also try to figure out whether the addition of angiogenesis inhibitors will bring better efficacy. Unfortunately, there was no statistically significant difference. As the numerical difference of mPFS between chemotherapies with or without angiogenesis exists, further studies may be of implementation value.

Different from chemotherapy, patients with RET fusion NSCLC have been unable to benefit well from MKI and ICI, and the same outcomes were shown in this study, though MKIs including cabozantinib and vandetanib were recommended in clinical guidelines (17–20). The expressions of PD-L1 in patients with RET fusion are heterogeneous, but most patients had a negative PD-L1 expression (21). The former study has proved that the expression of PD-L1 cannot affect the treatment efficacy in this group of patients (22). The same as the former study, the PD-L1 expression level did not correlate with the efficacy of ICI in patients with RET-fusion NSCLC in our result. Besides, the mPFS of ICI-based regimens was 6.7 months which seems longer than the one in a former study (17). This result may be caused by the use of immune-combination therapy in most patients, which may bring better efficacy than ICI monotherapy, and its efficacy is not directly correlated with the expression of PD-L1. In former studies, the mPFS of MKI in patients with RET-fusion NSCLC ranged from 3.4 to 7.3 months with poor tolerability due to off-targeted activity (23). Although safety analysis was not performed in this study, the efficacy of MKI was also poor as in previous studies, further suggesting the importance of precise targeting.

There are still some limitations of this study. First, this is a multicenter retrospective study, which means that bias was inevitable in the data collection process and some data were missing. Second, the number of patients in this study is not large enough, which prevented some more detailed subgroup analyses from being completed. Moreover, for the reason that we lack the records of adverse events, the safety analysis among different regimens cannot be achieved.

In conclusion, RET-TKI is the best choice for patients with RET-fusion-positive NSCLC nowadays, and chemotherapy is still a good choice. Besides, ICI-based regimens and MKI should not be recommended for this group of patients.

## Data Availability Statement

The raw data supporting the conclusions of this article will be made available by the authors, without undue reservation.

## Author Contributions

YM, YY, and YF designed the study. XL, XX, HD, JW, MZ, and NS collected the patients’ data. YM, YY, YF, ZX, ML, and MO analyzed the data. YY, YF, YQ, CS, and MZ drafted and revised the manuscript. All authors contributed to the article and approved the submitted version.

## Funding

This study is supported by State Key Laboratory of Respiratory Disease-The open project [SKLRD-OP-202111], Beijing Xisike Clinical Oncology Research Foundation [Y-XD2019-136], and Beijing Xisike Clinical Oncology Research Foundation [Y-2019Genecast-076].

## Conflict of Interest

The authors declare that the research was conducted in the absence of any commercial or financial relationships that could be construed as a potential conflict of interest.

## Publisher’s Note

All claims expressed in this article are solely those of the authors and do not necessarily represent those of their affiliated organizations, or those of the publisher, the editors and the reviewers. Any product that may be evaluated in this article, or claim that may be made by its manufacturer, is not guaranteed or endorsed by the publisher.
