# Supplementary material for: The Treatment Status of Patients in NSCLC With RET Fusion Under the Prelude of Selective RET-TKI Application in China: A Multicenter Retrospective Research
Source: Front Oncol. 2022 May 24;12:864367. doi: 10.3389/fonc.2022.864367 (PMC9176213; doi:10.3389/fonc.2022.864367)
Supplement: Supplementary file 1 [file DataSheet_1.docx]

**Supplementary Tables**

**Table S1 The p-value of pairwise comparison among different treatments in Log-rank test or Breslow test on PFS.**

| Log-rank |  | RET-TKI | Chemotherapy | ICI-based regimens | MKI | Breslow |
| --- | --- | --- | --- | --- | --- | --- |
|  | RET-TKI |  | 0.212 | 0.001* | ＜0.001* |  |
|  | Chemotherapy | 0.096 |  | 0.008^#*^ | ＜0.001* |  |
|  | ICI-based regimens | ＜0.001* | 0.013 |  | 0.096 |  |
|  | MKI | ＜0.001* | ＜0.001* | 0.040 |  |  |

*p＜0.008. After Bonferroni correction, p-value＜0.008 is considered statistically significant.

#The p-value between chemotherapy and ICI-based regimens was 0.0076 (＜0.008).

RET-TKI, rearranged during transfection-tyrosine kinase inhibitors. ICI, immune-checkpoint inhibitor. MKI, multi-kinase inhibitor.

**Table S2 The p-value of pairwise comparison among different treatments in different lines in Log-rank test**

| First line |  | RET-TKI |  |  |
| --- | --- | --- | --- | --- |
|  | RET-TKI |  | Chemotherapy |  |
|  | Chemotherapy | 0.056 |  | ICI-based regimens |
|  | ICI-based regimens | 0.019 | 0.527 |  |
| Subsequent line | RET-TKI |  |  |  |
|  | Chemotherapy | 0.230 |  |  |
|  | ICI-based regimens | 0.001* | 0.004* |  |

*p＜0.017. After Bonferroni correction, p-value＜0.017 is considered statistically significant.

RET-TKI, rearranged during transfection-tyrosine kinase inhibitors. ICI, immune-checkpoint inhibitor.
